# The spatio-temporal expression analysis of parathyroid hormone like hormone gene provides a new insight for bone growth of the antler tip tissue in sika deer

**DOI:** 10.5713/ab.23.0421

**Published:** 2024-02-28

**Authors:** Haihua Xing, Ruobing Han, Qianghui Wang, Zihui Sun, Heping Li

**Affiliations:** 1 College of Wildlife and Protected Area, Northeast Forestry University, Harbin 150040, China

**Keywords:** Antlers, Characterization, Cloning, Gene Expression, Sika Deer

## Abstract

**Objective:**

Parathyroid hormone like hormone (*PTHLH*), as an essential factor for bone growth, is involved in a variety of physiological processes. The aim of this study was to explore the role of *PTHLH* gene in the growth of antlers.

**Methods:**

The coding sequence (CDS) of *PTHLH* gene cDNA was obtained by cloning in sika deer (*Cervus nippon*), and the bioinformatics was analyzed. The quantitative real-time polymerase chain reaction (qRT-PCR) was used to analyze the differences expression of *PTHLH* mRNA in different tissues of the antler tip at different growth periods (early period, EP; middle period, MP; late period, LP).

**Results:**

The CDS of *PTHLH* gene was 534 bp in length and encoded 177 amino acids. Predictive analysis results revealed that the PTHLH protein was a hydrophilic protein without transmembrane structure, with its secondary structure consisting mainly of random coil. The PTHLH protein of sika deer had the identity of 98.31%, 96.82%, 96.05%, and 94.92% with *Cervus canadensis*, *Bos mutus*, *Oryx dammah* and *Budorcas taxicolor*, which were highly conserved among the artiodactyls. The qRT-PCR results showed that *PTHLH* mRNA had a unique spatio-temporal expression pattern in antlers. In the dermis, precartilage, and cartilage tissues, the expression of *PTHLH* mRNA was extremely significantly higher in MP than in EP, LP (p<0.01). In the mesenchyme tissue, the expression of *PTHLH* mRNA in MP was significantly higher than that of EP (p<0.05), but extremely significantly lower than that of LP (p<0.01). The expression of *PTHLH* mRNA in antler tip tissues at all growth periods had approximately the same trend, that is, from distal to basal, it was first down-regulated from the dermis to the mesenchyme and then continuously up-regulated to the cartilage tissue.

**Conclusion:**

*PTHLH* gene may promote the rapid growth of antler mainly through its extensive regulatory effect on the antler tip tissue.

## INTRODUCTION

Antlers of Cervidae are the only mammalian appendage that is capable of periodic shedding and complete regeneration and development, which is significantly different from the osseous cranial appendages of Antilocapridae, Bovidae, Giraffidae and others [1,2]. Antlers can grow up to 30 kg at a rate of 1 to 2 cm/d during the rapid growth period, exceeding the proliferation rate of some cancer cells, which are mainly regulated by the antler growth center (AGC) [3]. The AGC is in the tip tissue composed of multiple tissue layers [4]. Studies have shown that antlers are rich in a large number of growth factors, transcription factors and extracellular matrix proteins, which form a complex regulatory network that acts on the whole process of antler growth, making them unique and an ideal model for the study of mammalian cell genesis, growth and differentiation [5–7].

PTHLH, also known as parathyroid hormone-related peptide (PTHrP), was initially identified as a tumor factor of humoral hypercalcemia of malignancy (HHM) and was later found to be expressed in a variety of tissue cells such as keratinocytes and follicles [8–10]. *In situ* hybridization results showed that PTHLH and its receptor genes were expressed in skeletal and skin tissues during fetal development of rats and showed tissue specificity over time [11]. *PTH/PTHLH* receptor mRNA was highly expressed in the skin of mice during hair development and significantly low in telogen, and it was localized in the inner root sheath (IRS) in anagen and catagen [12]. It indicated that the *PTHLH* gene may play a role in the growth of skin and hair. PTHLH is also an important growth factor that regulates cartilage growth, and PTHLH promoted chondrogenesis as well as hypertrophy inhibition *in vitro* in bone marrow-derived MSCs (BMMSCs) and adipose tissue-derived MSCs (ATMSCs) [13,14]. Adding exogenous PTHLH at the early stage of chondrogenesis can differentiate MSCs into more chondrocytes and reduce the production of hypertrophic markers [15]. After targeted destruction of the *PTHLH* gene, the mouse rib cartilage consists of more hypertrophic chondrocytes, which is different from normal bone development, suggesting that the deletion of the *PTHLH* gene can alter the process of endochondral ossification and the development of chondrocyte. At the same time, the absence of *PTHLH* resulted in significant shrinkage of growth plates, especially in rapidly growing bones [16]. These studies suggest that PTHLH is an essential factor for normal cartilage growth and regulates the rate of chondrocyte differentiation.

Moreover, PTHLH and its receptor were expressed in antler tip tissues of red deer (*Cervus elaphus*) with tissue variability, and they were detected as hub genes regulating rapid antler growth and chondrogenesis using co-expression network analysis, suggesting that the *PTHLH* gene may have a complex regulatory role in antlers, so it is necessary to explore the function mechanism of *PTHLH* gene in antlers [17,18]. However, the structure of *PTHLH* gene and its spatio-temporal expression pattern are still unclear in sika deer antler and need to be further explored. And at present, there are no studies related to the spatio-temporal expression of genes within antlers. Based on this, different tissue layers of antler tip at three growth periods (early period, EP; middle period, MP; late period, LP) of sika deer, a representative animal of the Cervidae, were selected as the research objects in this study. The complete coding sequence (CDS) of *PTHLH* gene cDNA was obtained for the first time by molecular cloning technique in sika deer, and the bioinformatics was analyzed and mined. Quantitative real-time polymerase chain reaction (qRT-PCR) was used to analyze the expression of *PTHLH* mRNA in different tissues of the antler tip at different growth periods, to reveal the spatio-temporal expression patterns of *PTHLH* gene and to investigate its biological role and intrinsic mechanism in the growth process of the antler, which is intended to provide new ideas and scientific references for studying the molecular mechanisms of antler growth regulation.

## MATERIALS AND METHODS

All animal handling and procedures involved in this study were approved by the Institutional Animal Care and Use Committee of Northeast Forestry University (Harbin, China) (2023056).

### Sample collection

All tissue materials were collected from 5-year-old adult male sika deer (n = 3) in Jindi Breeding Deer Farm (Harbin, China). When the antler grew to EP (30 d), MP (60 d) and LP (90 d), the antler tip tissue was collected and the dermis, mesenchyme, precartilage and cartilage tissue layers were separated. And 36 samples were frozen in liquid nitrogen for subsequent experiments.

### Total RNA extraction and cDNA synthesis

The samples were crushed, and total RNA was extracted according to the steps of the column animal RNAout kit (TIANDZ, Beijing, China). Reverse transcription was performed with the PrimeScript RT Reagent Kit with gDNA Eraser (Perfect Real Time) (TaKaRa, Dalian, China). The 10.0 μL reaction system for genomic DNA removal contained 2.0 μL 5×gDNA Eraser Buffer, 1.0 μL gDNA Eraser, 2.0 μL total RNA, 5.0 μL ddH_2_O. The PCR conditions were 42°C for 2 min. The 20.0 μL reaction system for cDNA synthesis contained 10.0 μL of the previous reaction solution, 1.0 μL Prime Script RT Enzyme Mix I, 1.0 μL RT Primer Mix, 4.0 μL 5×Prime Script Buffer 2, 4.0 μL ddH_2_O. The PCR conditions were 37°C for 15 min, 85°C for 5 s. The synthesized cDNA was stored at −20°C for backup.

### *PTHLH* gene cloning

The primers were designed with Oligo7 software by referring to the *PTHLH* mRNA sequences of cattle (*Bos taurus*; accession number, NM_174753.1) in homologous species in the Genbank database (F: 5′-CAGAGCGAGAGGATAC GATG-3′, R: 5′-ACATGGTTCATTATTACAGAATCCT-3′). The PCR amplification was performed in a total volume of 20.0 μL reaction fluid containing 2.0 μL cDNA, 0.8 μL each primer, 10.0 μL 2×Rapid Taq Master Mix, 6.4 μL ddH_2_O. The PCR conditions for *PTHLH* were 95°C for 5 min, 40 cycles at 95°C for 30 s, 59.5°C for 30 s, and 72°C for 1 min and then a final extension at 72°C for 10 min. The amplification products were recovered and purified by TaKaRa MiniBEST Agarose Gel DNA Extraction Kit (TaKaRa, China) and ligated with pMD18-T vector. After the ligation, the products were transformed into Escherichia coli DH5α Competent Cells, spread on LB solid medium, and incubated at 37°C for 14 h. A single white colony was selected and transferred to LB liquid medium supplemented with ampicillin for shaking incubation. PCR amplification and 1% agarose gel electrophoresis were performed, where the maker used was DL2000 Plus DNA Marker (Vazyme, Nanjing, China), and the positive results were bidirectional sequencing.

### Bioinformatics analysis

The peak plots of sequencing results were viewed and calibrated with DNAStar software to obtain accurate cDNA sequences, coding sequences, and then translated into amino acid sequences using DNAMAN. The physicochemical properties, hydrophilicity/hydrophobicity, and tertiary structure of PTHLH protein were predicted using Protparam, ProtScale, and Swiss-Model tools in ExPASy online server (https://www.expasy.org). The transmembrane structure and secondary structure of PTHLH protein were predicted and analyzed using TMHMM-2.0 Server (https://services.healthtech.dtu.dk/services/TMHMM-2.0/), SOPMA tool in NPSA online server (https://npsa-prabi.ibcp.fr/cgi-bin/npsa_automat.pl?page=/NPSA/npsa_server.html), and the signal peptide, phosphorylation sites and subcellular localization of PTHLH protein were predicted using SignalP-6.0 Server (https://services.healthtech.dtu.dk/services/SignalP-6.0), Netphos-3.1 Server (https://services.healthtech.dtu.dk/services/NetPhos-3.1) and PSORT II Server (https://psort.hgc.jp/form2.html). The amino acid sequences were verified by Blastp (https://www.ncbi.nlm.nih.gov/BLAST) for comparison and a preliminary analysis of the identity of the PTHLH gene between sika deer and other species. The amino acid sequences of PTHLH proteins of other 11 species were downloaded on NCBI (https://www.ncbi.nlm.nih.gov), and the identity of PTHLH amino acid sequence among different species was further analyzed using the BioEdit software. The species names and GenBank accession numbers of the PTHLH sequences were as follows: cattle (Accession number, AAI49412.1), dingo (*Canis lupus dingo*; accession number, XP_035563257.1), red deer (Accession number, XP_043738180.1), cat (*Felis catus*; accession number, XP_ 019690307.1), wolverine (*Gulo gulo luscus*; accession number, KAI5769541.1), human (*Homo sapiens*; accession number, CAG46680.1), white-tailed deer (*Odocoileus virginianus texanus*; accession number, XP_020 766534.1), sheep (*Ovis aries*; accession number, XP_04210 3283.1), orangutan (*Pongo abelii*; accession number, XP_ 009245858.2), leopard (*Panthera uncia*; accession number, XP_049481166.1), pig (*Sus scrofa*; accession number, AAO 39324.2).

### qRT-PCR of *PTHLH* mRNA in sika deer antler tip tissues at different growth periods

The level of *PTHLH* gene transcription was detected by ABI 7500 Real-time system (Applied Biosystems, CA, USA). The qRT-PCR experiments were performed according to the instructions of TB Green Premix Ex Taq II (Tli RNase H Plus) (TaKaRa, China). The primers were designed according to the cDNA sequence of *PTHLH* gene in sika deer (F: 5′-CC GCTTTGGGTCTGATG-3′, R: 5′-GAGTTCGCCGTTTC TTCTT-3′), and *β-actin* was used as the internal reference gene (F: 5′-GCGTGACATCAAGGAGAAGC-3′, R: 5′-GGA AGGACGGCTGGAAGA-3′). For each of the 36 samples, three replicate groups were designed for *PTHLH* and internal the reference gene.

### Statistical analysis

Gene expression data were analyzed using the 2^−ΔΔCt^ method and statistical analysis was performed by one-way analysis of variance and Tukey test and using IBM SPSS Statistics 23.0 software. Data represent mean±standard deviation. Statistical significance is defined when p values are less than 0.05, and 0.01<p<0.05, p<0.01 indicate significant difference and extremely significant difference, respectively.

## RESULTS

### cDNA cloning and positive detection of *PTHLH* gene

**Figure 1:**
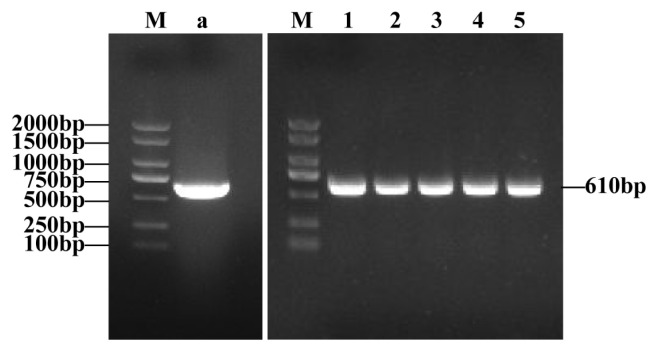
Polymerase chain reaction amplification results of *PTHLH* gene. M, DL2000 Plus DNA Marker. a, amplification product. 1 to 5, positive detection results of cloning products. *PTHLH*, parathyroid hormone like hormone.

The PCR amplification with positive detection of cloned products showed that a single bright band of 610 bp was obtained, which was consistent with the length of the expected fragment (Figure 1). The 610 bp sequence obtained by sequencing was compared with BLAST showing that the sequence was highly identical to the *PTHLH* gene of red deer (Accession number, AY328402.1), cattle (Accession number, NM_174753.1), takin (Accession number, XM_ 052640243.1) and goat (*Capra hircus*; accession number, NM_001285753.1), confirming this sequence as the cDNA sequence of the *PTHLH* gene of sika deer.

### Results of bioinformatics analysis of the *PTHLH* gene of sika deer

**Figure 2:**
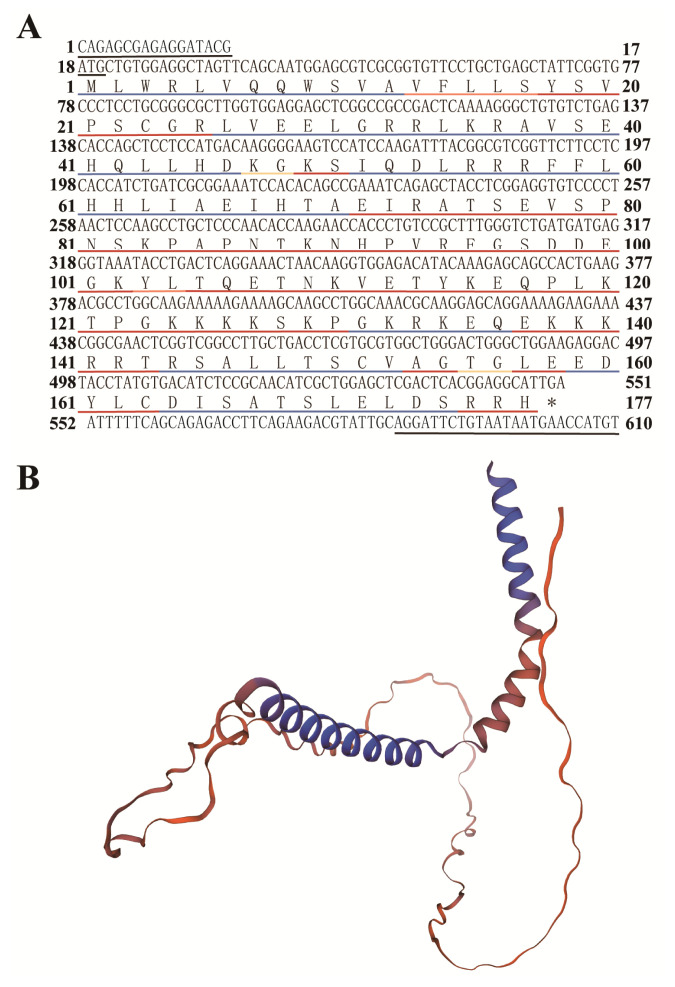
Structure of the encoded protein of the PTHLH gene in sika deer. (A) Nucleotide sequence and deduced amino acid sequence and secondary structure of *PTHLH* gene. * stop codon. (B) Tertiary structure of PTHLH protein. *PTHLH*, parathyroid hormone like hormone.

The predicted physicochemical properties of the encoded proteins of *PTHLH* gene of sika deer showed that the CDS was 534 bp in length and encoded a total of 177 amino acids (Figure 2A), of which Leu was the most abundant with 20, accounting for 11.3% of all amino acids, followed by Lys with 19, accounting for 10.7% of all amino acids. It had a relative molecular weight of 20,318.27, theoretical pI of 9.83 and aliphatic index of 76.55. The molecular formula was C_891_H_1457_N_271_O_264_S_4_, with a total atomic number of 2,887. The instability index of PTHLH proteins was 61.97 and the grand average of hydropathicity (GRAVY) was −0.851, indicating that the protein was an unstable hydrophilic protein. The prediction result of secondary structure showed that the PTHLH protein had 86 random coil, 79 α-helix, 7 extended strand, and 5 β-turn, accounting for 48.59%, 44.63%, 3.95%, and 2.82% of all proteins, respectively (Figure 2A). The result of protein structure homology-modelling in Swiss-Model showed that the amino acid sequence of PTHLH protein had 97.44% identity and 60.00% similarity to the template, indicating that the tertiary structure was more accurate (Figure 2B).

**Figure 3:**
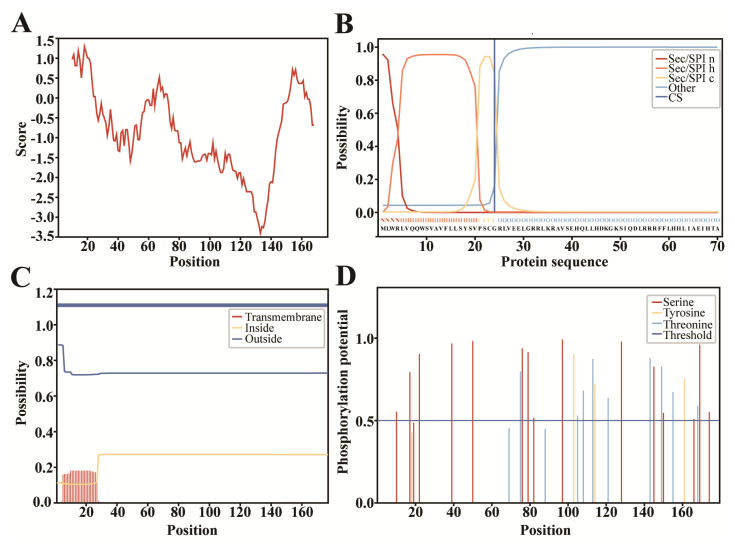
Bioinformatics prediction results of PTHLH protein. (A) Prediction results of hydrophilicity/hydrophobicity. (B) Prediction results of signal peptide. Sec/SPI represented secretory signal peptides transported by the Sec translocon and cleaved by Signal Peptidase I. Sec/SPI n, Sec/SPI h and Sec/SPI c represented the region structure of the Sec/SPI. CS, Cleavage site. (C) Prediction results of transmembrane structure. (D) Prediction results of phosphorylation sites. PTHLH, parathyroid hormone like hormone.

The prediction result by Kyte&Doolittle method showed that the PTHLH protein was a hydrophilic protein, which was consistent with the prediction result of Protparam (Figure 3A). The PTHLH protein had a signal peptide, and the probability of a cleavage site between amino acids 24 and 25 was 0.8440 (Figure 3B). In addition, it did not have transmembrane structure (Figure 3C). The prediction result of phosphorylation sites showed that the PTHLH protein may contain phosphorylation sites for kinases such as protein kinase A (PKA), protein kinase C (PKC), protein kinase G (PKG) and p38 mitogen-activated protein kinase (p38 MAPK), with 15 serine, 9 threonine, and 3 tyrosine phosphorylation sites, respectively (Figure 3D). The results of subcellular localization showed that the PTHLH protein was localized in the nucleus with the highest probability of 73.9%, in the mitochondria with 13.0%, in the cytoplasm with 8.7% and in the plasma membrane with 4.3%.

The deduced amino acid sequences were compared by Blastp which showed that the PTHLH protein of sika deer had the highest identity of 98.31% with elk (Accession number, XP_043297243.1), and 96.82%, 96.05%, and 94.92% with other species such as wild yak (Accession number, ELR57204.1), scimitar-horned oryx (Accession number, XP_040090551.1) and takin (Accession number, XP_052496203.1), respectively. Further alignment results revealed that the PTHLH protein of sika deer exhibited the highest identity with artiodactyls, including red deer, white-tailed deer, cattle, and pig. Additionally, it demonstrated a high identity with carnivores such as cat and leopard but showed a lower identity with primates (Table 1).

### Differential analysis of *PTHLH* mRNA expression in sika deer antler tip tissues at different growth periods

**Figure 4:**
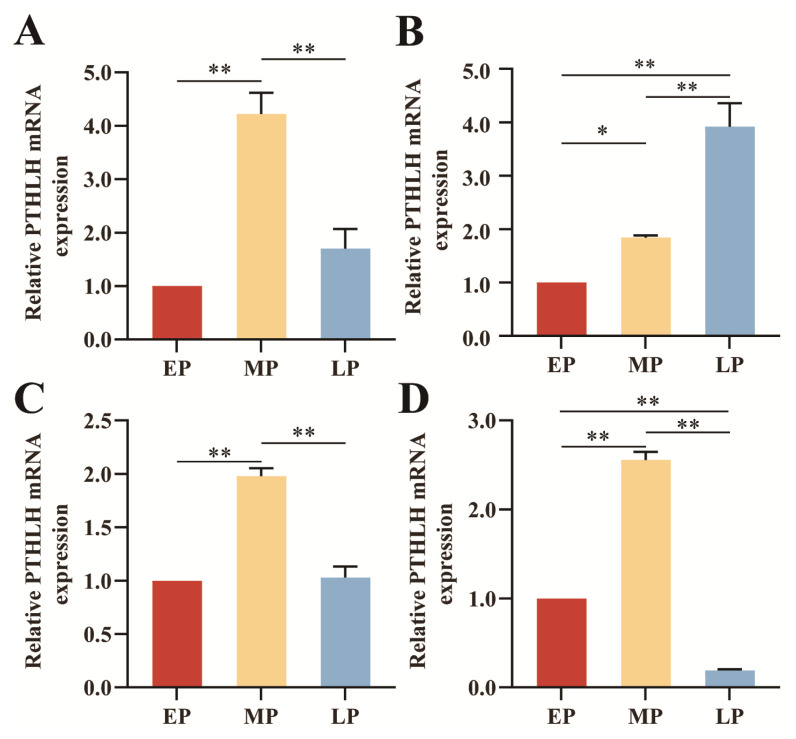
Differential expression of *PTHLH* mRNA in different tissues. (A) Dermis tissue. (B) Mesenchyme tissue. (C) Precartilage tissue. (D) Cartilage tissue. EP, early period. MP, middle period. LP, late period. *PTHLH*, parathyroid hormone like hormone. * 0.01<p<0.05. ** p<0.01.

**Figure 5:**
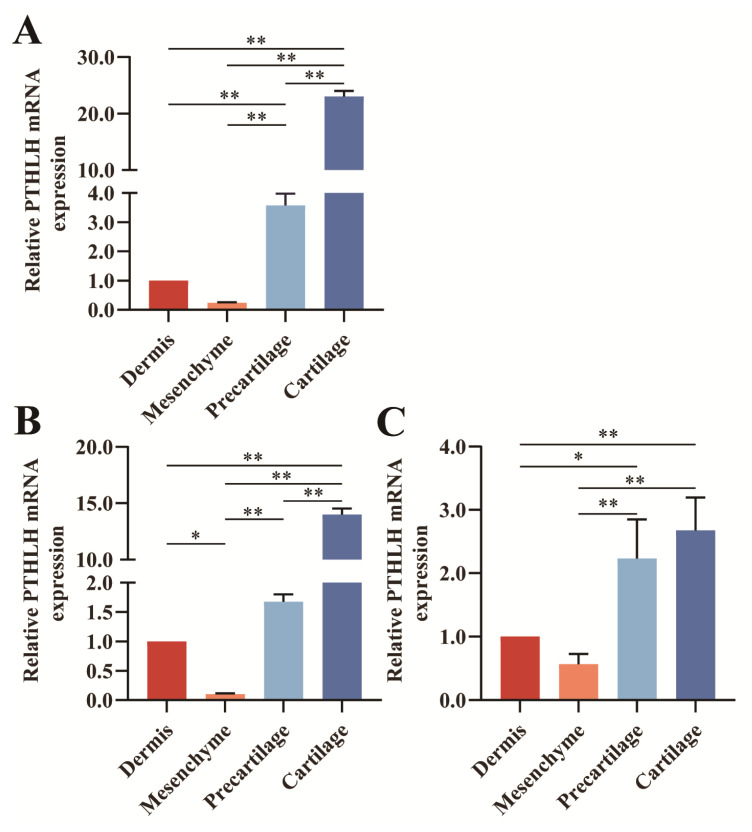
Differential expression of *PTHLH* mRNA at different growth periods. (A) Early period. (B) Middle period. (C) Late period. *PTHLH*, parathyroid hormone like hormone. * 0.01<p<0.05. ** p<0.01.

The qRT-PCR results showed that *PTHLH* mRNA was expressed in all tissues of sika deer antler tip at different growth periods, with spatio-temporal differential expression characteristics (Figure 4, 5). In the dermis, precartilage, and cartilage tissues, the expression of *PTHLH* mRNA was extremely significantly higher in MP (4.2213, 1.9794, 2.5580) than in EP (1.0000, 1.0000, 1.0000), LP (1.7029, 1.0293, 0.1891) (p<0.01), and *PTHLH* mRNA expression in MP (1.8421) was significantly higher than that of EP (1.0000) (p<0.05), but extremely significantly lower than that of LP (3.9190) in the mesenchyme tissue (p<0.01) (Figure 4). The expression of *PTHLH* mRNA in sika deer antler tissues at all growth periods had approximately the same trend, that is, from distal to basal, it was first down-regulated from the dermis to the mesenchyme and then continuously up-regulated to the cartilage tissue (Figure 5). It was highly expressed mainly in the precartilage and cartilage tissues.

## DISCUSSION

The unique growth and development characteristics of antler are closely related to its internal gene regulation. Many genes related to antler growth have been screened, but the internal mechanism of their role is still a mystery, which needs to be further explored [19]. The *PTHLH* gene is related to cartilage formation, angiogenesis, and cell proliferation and differentiation, speculating that it may be involved in and regulate the growth of antlers [20,21]. The focus of this study was to explore the expression of *PTHLH* gene in different tissues of the sika deer antler tip at different growth periods, to reveal its role in the growth and development of antlers.

In this study, we successfully cloned the CDS of the *PTHLH* gene of sika deer, with a fragment length of 534 bp, encoding 177 amino acids, and the secondary structure of its encoded protein was mainly random coil. The prediction result of phosphorylation sites showed that the PTHLH protein may contain phosphorylation sites for protein kinases such as PKA, PKC, PKG, and p38 MAPK. Protein phosphorylation, as a widespread reversible post-translational modification in biology, is involved in gene expression regulation, signal transduction, and is closely related to cell proliferation, apoptosis and development of cancer [22–25]. Inhibitors of p38 MAPK, PKC can reduce synthesis and secretion of PTHLH, speculating that PTHLH protein may be affected by the comprehensive action of various kinases to influence protein phosphorylation and its activity, thereby regulating the expression of the *PTHLH* gene and its biological role in the growth of antlers [26]. Comparison of amino acid homology revealed the highest homology between the *PTHLH* gene of sika deer and red deer. As can be seen from Table 1, the PTHLH protein of sika deer was most closely related to those of artiodactyls and more distantly related to those of the primates, which is consistent with the direction of evolution, and suggests that PTHLH protein is highly conserved in artiodactyls and may perform a similar function.

The expression level of *PTHLH* mRNA in antlers was investigated. The results showed that the *PTHLH* gene was expressed in the dermis, mesenchyme, precartilage and cartilage tissues of the antler tip at different growth periods, and the expression amount differed with a unique spatio-temporal expression pattern.

The immunolocalization of red deer antler showed that *PTHLH* was obviously stained in the epidermis and cartilage tissue [17]. The results of the present study showed that *PTHLH* expression was down-regulated from dermis to mesenchyme tissue and consistently up-regulated from mesenchyme to cartilage tissue in sika deer antlers under all growth periods, suggesting that the *PTHLH* gene may have similar expression patterns and regulatory effects in antlers. In addition, PTHLH was highly expressed in precartilage and cartilage tissues in EP, MP, LP, and the differences with mesenchyme tissue were all extremely significant (p< 0.01), indicating that *PTHLH* plays an important role in the development of antler bone tissue. In the dermis, precartilage, and cartilage tissues, the expression of *PTHLH* mRNA was extremely significantly higher in MP than in EP, LP (p<0.01), indicating that *PTHLH* may play a positive role in regulating the rapid growth of antler.

In the dermis, the expression of *PTHLH* mRNA showed a trend of increasing and then decreasing as growth and development progressed, indicating that *PTHLH* promotes the rapid growth of dermis. The skin of antlers is composed of epidermis and dermis and has abundant hair follicles and sebaceous glands [27,28]. The expression of *PTHLH* and its receptor mRNA in dermal fibroblasts indicated that *PTHLH* may interact with each other through autocrine signaling to regulate the growth of antler dermis [17,29]. It is worth noting that dermal fibroblasts have the properties of promoting wound healing, indicating that *PTHLH* may also have a potential effect on the physiological function of fibroblasts, and thus guide rapid growth of antler skin [30].

The expression level of *PTHLH* mRNA in mesenchyme tissues was lower than that in other tissues at the same time, which was also observed in red deer antlers [17]. Antler regeneration is stem cell-dependent, and during the growth and development of antlers, antler stem cells are successively differentiated into mesenchymal cells, precartilage cells and chondrocytes [31–34]. *PTHLH* can enhance the osteogenic differentiation ability of MSCs, and promote the expressions of Runx2, Sp7 mRNA and OCN protein, which are the regulatory factors of osteogenic process [35]. In addition, *PTHLH* mRNA expression was consistently up-regulated in antler mesenchyme tissues with growth and development, suggesting that *PTHLH* may play a positive role in the differentiation and maturation of antler MSCs.

Immunohistochemical analysis showed that recently differentiated chondrocytes had expression of PTHLH protein, and the results of this study further confirmed that *PTHLH* mRNA was highly expressed in precartilage and cartilage tissues and had the same expression pattern, that is, the expression increased and then decreased during the growth period of antlers [27]. Deletion of *PTHLH* gene can slow down the proliferation of chondrocytes and accelerate the maturation of chondrocytes, suggesting that *PTHLH* may be an essential factor for the normal development of chondrocytes and maintain the normal growth of cartilage tissues [16].

The addition of *PTHLH* can increase the expression of cyclin D1 mRNA in chondrocytes, and the addition of PKA and PKC inhibitors in the downstream signaling pathways of *PTHLH* can effectively change the stimulation of cyclin D1 [29]. Both Matrix metalloproteinase-9 (*MMP9*) and Matrix metalloproteinase-13 (*MMP13*) can be detected in antler chondrocytes [36]. MMP13 is a marker of chondrocyte hypertrophy and acts cooperatively with MMP9 to degrade cartilage extracellular matrix [36,37]. p38MAPK, PKC and c-Jun N-terminal kinase (JNK) signaling pathway inhibitors can weaken the expression inhibition of MMP13 and MMP9 by PTHLH, respectively [36]. The above indicated that *PTHLH* can regulate the expression level of downstream genes by various signaling pathways and promote the rapid growth of antler bone tissue by regulating the proliferation of antler chondrocytes and matrix degradation. Antler osteoclasts in bone tissue are the basis of tissue remodeling, and *PTHLH* can promote the differentiation of osteoclast-like multinucleated cells into osteoblasts [38,39]. It is hypothesized that *PTHLH* may also regulate the efficiency of cartilage tissue remodeling of antlers by directing the generation of osteoclasts, thereby promoting the rapid growth of antlers.

This study, for the first time at the transcriptional level, revealed the spatio-temporal expression pattern of *PTHLH* in sika deer antlers, which provides foundational data for the research on the internal regulatory mechanism of antler growth, development and regeneration, and serves as a scientific reference for the study of bone diseases and fracture healing in humans, mice and others. In addition, we will further investigate the expression levels of the PTHLH protein in deer antlers to explore the function of the *PTHLH* gene.

## CONCLUSION

This study identified the biological information of *PTHLH* gene in sika deer and the unique spatio-temporal expression pattern of *PTHLH* mRNA in the antler tip tissues and speculated that *PTHLH* may have different physiological functions in antler tip tissues at different growth periods, but mainly promotes the bone growth of the antler tip tissues through a variety of precise regulation. Subsequently, further investigation can be undertaken on the epigenetic regulation of the *PTHLH* gene and the spatio-temporal expression of the protein in the antler tip tissues, aiming for a more comprehensive understanding of the intrinsic mechanisms underlying the growth and development of antlers.

## Figures and Tables

**Table 1:** The identity of PTHLH amino acid sequence among different species (%)

Species	Sika deer	Red deer	White-tailed deer	Human	Orangutan	Wolverine	Cattle	Pig	Sheep	Leopard	Cat	Dingo
Sika deer	-	98.3	96.0	89.2	88.1	90.3	97.1	90.3	95.4	92.0	92.0	90.9
Red deer	98.3	-	97.7	89.8	89.8	90.9	97.1	90.3	96.0	92.6	92.6	91.5
White-tailed deer	96.0	97.7	-	90.3	90.3	92.0	97.7	91.5	97.1	92.6	92.6	92.6
Human	89.2	89.8	90.3	-	98.3	92.0	90.3	90.9	89.8	92.0	92.0	91.5
Orangutan	88.1	89.8	90.3	98.3	-	90.3	89.2	89.8	88.7	90.9	90.9	90.3
Wolverine	90.3	90.9	92.0	92.0	90.3	-	92.0	93.2	90.9	97.1	97.1	97.1
Cattle	97.1	97.1	97.7	90.3	89.2	92.0	-	92.6	97.7	93.2	93.2	92.6
Pig	90.3	90.3	91.5	90.9	89.8	93.2	92.6	-	91.5	93.2	93.2	92.6
Sheep	95.4	96.0	97.1	89.8	88.7	90.9	97.7	91.5	-	92.0	92.0	91.5
Leopard	92.0	92.6	92.6	92.0	90.9	97.1	93.2	93.2	92.0	-	100.0	97.7
Cat	92.0	92.6	92.6	92.0	90.9	97.1	93.2	93.2	92.0	100.0	-	97.7
Dingo	90.9	91.5	92.6	91.5	90.3	97.1	92.6	92.6	91.5	97.7	97.7	-

PTHLH, parathyroid hormone like hormone.

## Data Availability

The data presented in this study are available on request from the corresponding author.
